# The effect of intentionality on verbal memory assessment over days

**DOI:** 10.1590/1980-57642020dn14-040006

**Published:** 2020-12

**Authors:** Renata Kochhann, Bárbara Costa Beber, Patrícia Ferreira, Maila Rossato Holz, Rafael Ruschel, Analuiza Camozzato de Pádua, Cláudia da Cunha Godinho, Iván Izquierdo, Márcia Lorena Fagundes Chaves

**Affiliations:** 1 Dementia Clinic, Neurology Service, Hospital de Clínicas de Porto Alegre - Porto Alegre, RS, Brazil.; 2 Research Projects Office, Hospital Moinhos de Vento - Porto Alegre, RS, Brazil.; 3 Universidade Federal de Ciências da Saúde de Porto Alegre - Porto Alegre, RS, Brazil.; 4 School of Health and Life Sciences, Pontifícia Universidade Católica do Rio Grande do Sul - Porto Alegre, RS, Brazil.; 5 University of East London - London, United Kingdom.; 6 Memory Center, Brain Institute, Pontifícia Universidade Católica do Rio Grande do Sul - Porto Alegre, RS, Brazil.

**Keywords:** aging, memory, episodic, intention, learning, cognition, envelhecimento, memória episódica, intenção, aprendizagem, cognição

## Abstract

**Background:**

Intentionality to remember is associated with better performances in episodic memory retrieval. The practice effect has better performance in memory retrieval. However, little is known about the effect of intentionality on memory over days and the influence of age, gender, and level of education on it as well as on practice effect.

**Objectives:**

To verify the effect of intentionality and practice effect on memory performance over days, using an ecological approach.

**Methods:**

One hundred and twenty subjects from 18 to 81 years of age and free of psychiatric and neurological disorders were evaluated. They were randomized into a “testing effect group” and a “intentionality group” and then were asked to read a text on the FIFA World Cup. The “intentionality group” was instructed to pay careful attention to the text because they would answer a questionnaire with 10 factual items from the text after 2 and 7 days. The “testing effect group” had the same procedure at the same time as the first group but were not instructed about the intentionality, and answered the questionnaire immediately after reading the text.

**Results:**

Memory performance was better 2 days after the exposure session than 7 days later in the “intentionality group”. On the other hand, there was no difference in memory performance from the “testing effect group” 2 and 7 days later.

**Conclusions:**

Intention to recall may enhance memory over a short period of days, while retaining similar amount of information over days to what was acquired immediately after text exposure.

## RESUMO

**Introdução:**

A intencionalidade de lembrar associa-se a melhores desempenhos na recuperação da memória episódica. O efeito da prática também apresenta melhores desempenhos na recuperação da memória. Entretanto, pouco se sabe sobre a intencionalidade na memória ao longo dos dias sob influência da idade, sexo e escolaridade, assim como sob efeito da prática.

**Objetivos:**

Verificar o efeito da intencionalidade e de testes no desempenho da memória ao longo dos dias, utilizando abordagem ecológica.

**Métodos:**

Foram avaliados 120 sujeitos (idade 18-81 anos) e a ausência de distúrbios psiquiátricos e neurológicos. Eles foram randomizados para o “grupo de efeito de testes” ou para o “grupo de intencionalidade” e expostos ao texto da Copa do Mundo de Futebol. O “grupo de intencionalidade” foi instruído a prestar muita atenção ao texto, pois seria aplicado um questionário com 10 itens factuais do texto 2 e 7 dias depois. O “grupo de efeito de testes” realizou o mesmo procedimento no mesmo tempo que o primeiro grupo, mas não foi instruído em relação à intencionalidade e responderam ao questionário imediatamente após a leitura do texto.

**Resultados:**

O desempenho da memória foi melhor 2 dias após a exposição do que 7 dias depois no “grupo de intencionalidade”. Por outro lado, não houve diferença no desempenho de memória no “grupo de efeito de testes” 2 e 7 dias depois.

**Conclusões:**

A intenção de recordar pode melhorar a memória por um curto período de dias. Enquanto o efeito de teste pode reter ao longo de dias quantidade similar de informação que foi adquirida imediatamente após a exposição do texto.

## INTRODUCTION

Intact cognitive abilities are crucial to perform everyday activities such as managing finances, remembering appointments, remembering medications, driving in unfamiliar places, remembering a grandchild's birthday, telling a story, and learning to use a new computer application. Human memory includes a declarative and a procedural system.^1^ Declarative memory refers to conscious and intentional memory for experiences (episodic memory) and concepts and facts (semantic memory).^1^ The episodic memory (part of the declarative system) is responsible for encoding, storing, and retrieving personal and autobiographical information.^2^ Episodic memory is one of the essential cognitive abilities that support remembering events and is especially vulnerable to the aging process.^3^ However, there has been an increase in concern about the memory capacity of young adults.

Finding strategies to enhance memory in the aged population is an old concern; however, there is an increasing interest in young individuals also due to the importance of memory for good academic and professional achievements.^4^ ^–^ ^6^ Evidence on the effectiveness of memory strategies is essential to define methods of assessment and neuropsychological rehabilitation strategies to better understand the neurobiology of learning and memory.

One strategy to enhance memory that has already been investigated is the testing effect, *i.e.*, the improvement of memory retention as a consequence of memory test. In a long-term retention period, 2 days and 1 week later, subjects who had taken an initial test recalled more information than subjects who had only studied the passages which covered a single topic.^7^ When all participants answered a final test, those who answered a test after studying a specific material perform better than the group that only restudied the material.^8^ A study that used a repetition measure over time states that the use of a test at the time of information acquisition helped the groups to improve more than 10% of their acquisition longitudinally in a real-life educational setting.^9^ Thus, assessment measures (*i.e*., testing) during acquisition are beneficial for storing information. However, it is still unclear whether they are enough for learning at different age strata.

On the other hand, another strategy to enhance memory that has already been investigated is the intentionality to remember, as episodic memories can be involuntary (usually unexpected) or voluntary.^10^ Learning and/or storing new information can be intentional (*i.e*., knowing that the information provided will be needed later) or incidental (*i.e*., not knowing that the information to be learned will be needed later).^10^ ^–^ ^12^ Incidental learning occurs naturally in the course of everyday events.^13^ Therefore, most of the declarative memories might be incidentally acquired.^14^ ^,^ ^15^ Intentional learning, on the other hand, has long been known for its positive effect on subsequent recall as compared to incidental learning.^16^

More recent studies have indicated that intentionality may be associated with better performances in memory retrieval. These studies used diverse paradigms and stimuli to test memory, *i.e.* faces and names,^17^ ^,^ ^18^ flavors,^19^ real objects,^20^ pictures,^21^ or words.^22^ To better understand the benefit of intentionality for memory, we consider important to highlight three caveats:

- some of the paradigms used in the previous studies may not resemble the demands faced by individuals in real-life situations;
- it is not known whether the effect of intentionality lasts for prolonged periods or not, as the research investigated immediate and recent/remote memory just by testing participants a few minutes or hours later;^18^ ^,^ ^21^ ^,^ ^22^
- episodic memory is affected by the aging process^3^ ^,^ ^4^ and previous research have found age-related deficits under intentional conditions of association memory tasks.^11^ ^,^ ^23^

In this way, it is important to investigate the effect of intentionality on more ecological tasks of verbal memory, and the use of the story recall task is one valid paradigm.^24^ ^,^ ^25^ It is also necessary to investigate the effect of intentionality over days with verbal tasks especially, since published information on intentionality over days are on memory for flavors (one and two days after the stimuli presentation session),^19^ or memory for real objects (tested after two days).^20^ Furthermore, the age effect on intentionality, using verbal logical memory tasks (or stories recall), has not been investigated until the moment. Based on the above caveats, the aim of this study was to verify the effects of intentionality and testing on memory performance over days, using an ecological approach. The influence of age, gender, and level of education on memory performance was also evaluated. Our first hypothesis was that intentionality would have a stronger influence on memory performance due to the motivation to remember, with a better effect on younger participants. The second hypothesis is that the testing effect will not show improvement as does the intentionality over time due to the stability of learning.

## METHODS

### Participants

One hundred and twenty subjects, functionally independent, with more than eight years of schooling and age ranging from 18 to 81 years were selected for the study. Participants did not present psychiatric and neurological disorders (scores less than eight in the Self-Reporting Questionnaire - SRQ),^26^ ^,^ ^27^ nor cognitive impairment in a screening test (scores higher than 24 in the Mini-Mental State Examination - MMSE).^28^ ^,^ ^29^ ^,^ ^30^ The participants were selected from non-patient individuals present in different sectors of the university hospital (patients’ relatives, visitors, and students). The project was approved by the institutional review board of Hospital de Clínicas de Porto Alegre (protocol number 100355) and all participants signed the informed consent form. This study comprised two samples and the participants were randomly distributed into the intentional learning group (ILG) (n=60) and the testing effect group (TEG) (n=60). The instruments were applied by specialists (neuropsychologists). The assessment of age effect included participants of large age variability (18 to 81 years old). Age and education were entered as continuous covariates in the analysis.

### Instruments

The assessment of formal episodic memory was carried out using the Formal Memory Task (FIFA World Cup text), a 15-line text with factual information on the 1954 FIFA World Cup.^31^ ^,^ ^32^ The participants were asked to study the text. Afterward, they answered a questionnaire with 10 factual items about information in the text.^31^ They were awarded one point for each correct answer; the maximum score was 10. The endpoint measure was the total correct score obtained in the questionnaire.

### Design and procedure

**Figure 1:**
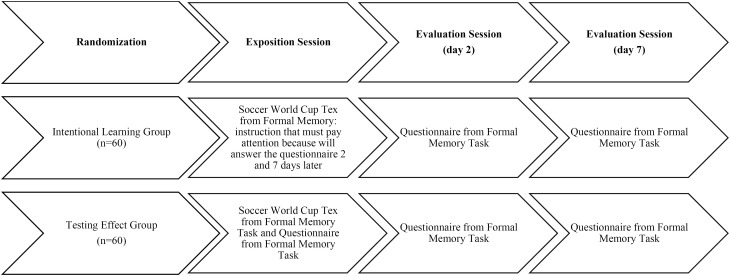
Study design.

After a presentation, subjects interested in this study obtained written informed consent from the staff. Participants were randomly assigned to the ILG (n=60) and the TEG (n=60), and were kept blind to the objectives of the study. In the ILG group, subjects read the FIFA World Cup text on day 1 and were informed that they should pay attention to the text in order to answer a questionnaire about it after 2 and 7 days. TEG participants read the text as the ILG group did, but they were not warned that this information would have to be recalled further. They were asked to respond to the questionnaire immediately after reading the text, two and seven days later. All subjects were given 10 min to read and study the text and all sessions were individually performed. Participants were not allowed to take notes of the information in the text. A telephone interview was used to administer the questionnaire on days 2 and 7 and the researchers who interviewed the participants were kept blind to the participant's group. Figure 1 shows the study design.

### Statistical analyses

Statistical analyses were performed using the IBM *Statistical Package for the Social Sciences* (SPSS) software (version 20.0 for Windows). Student's *t* test (for independent samples) was performed to compare the performance of episodic memory between genders. To determine whether memory performance differed between days, repeated measures analyses of variance (ANOVA) were carried out using the performance of the Formal Memory Task as the dependent variable. Age, gender, and education were used as covariates. Significance level was set at p£0.05.

## RESULTS

### Sociodemographic results

Demographic data are presented in Table 1.

**Table 1:** Demographic and cognitive data of participants.

	ILG (n=60)	TEG (n=60)
Age M (SD)/range	48.9 (16.2)/18-73	45.0 (18.4)/19-81
Education in years M (SD)/range	12.9 (2.32)/9-21	14.6 (3.58)/9-24
Gender - female N (%)	31 (51.7%)	41 (68.3%)
MMSE scores M (SD)/range	28.6 (1.36)/26-30	28.8 (1.56)/25-30

M: mean; N: absolut frequency; SD: standard deviation; ILG: intentional learning group; TEG: testing effect group; MMSE: Mini-Mental State Examination.

Performances over the days are presented in Table 2.

**Table 2:** Performance of the participants over the days in each group.

	ILG	TEG
Day 1 - all participants	–	5.45 (2.28)
Day 2 - all participants	6.53 (1.47)	4.68 (2.13)
Day 7 - all participants	3.80 (2.01)	4.52 (2.08)
Day 1 - females	–	5.02 (2.22)
Day 2 - females	6.10 (1.70)	4.12 (1.96)
Day 7 - females	3.65 (1.84)	4.12 (1.98)
Day 1 - males	–	6.37 (2.22)
Day 2 - males	7.00 (1.00)	5.89 (2.02)
Day 7 - males	3.97 (2.19)	5.37 (2.11)

ILG: intentional learning group; TEG: testing effect group.

### Within-subjects results - intentional learning group

Participants remembered more on day 2 than on day 7 (*F*(1.56)=5.94, p=.018; η_p_ ^2^=.096). There was no interaction between the factor (performance on days 2 and 7) and age (*F*(1.56)=0.03, p=.867; η_p_ ^2^=.001), gender (*F*(1.56)=1.06, p=.306; η_p_ ^2^=.019) or education (*F*(1.56)=0.45, p=.503; η_p_ ^2^=.008).

### Between-subjects results - intentional learning group

Education (*F*(1.56)=6.18, p=.016; η_p_ ^2^=.100) and gender showed statistically significant differences (*F*(1.56)=4.52, p=.038; η_p_ ^2^=.075). Student's *t*-test indicated that men presented better episodic memory at day 2 than women (*t*(58)=2.48, *p*=.015, *d*=-.64) but no difference was found in performance between men and women on day 7 (*t*(58)=1.76, *p*=.541, *d*=-.159).

### Within-subjects results - testing effect group

There were no differences in performance on days 1, 2, and 7 (*F*(1.56)=0.22, p=.641; η_p_ ^2^=.004). There was no interaction between the factor (performance on days 2 and 7) and age (*F*(1.56)=0.56, p=.455; η_p_ ^2^=.010), gender (*F*(1.56)=0.07, p=.795; η_p_ ^2^=.001) or education (*F*(1.56)=0.01, p=.919; η_p_ ^2^=.000).

### Between-subjects results - testing effect group

Only gender showed statistically significant differences (*F*(1.56)=7.41, p=.009; η_p_ ^2^=.117). The Student's *t*-test indicated that men presented better episodic memory at days 1, (*t*(58)=2.18, *p*=.036, *d*=-.609), 2 (*t*(58)=3.22, *p*=.002, *d*=-.892), and 7 (*t*(58)=2.22, *p*=.030, *d*=-.619) than women.

## DISCUSSION

Our purpose in this study was to verify the effect of intentionality on remembering information, as well as the testing effect, two and seven days after the encoding, and applying a naturalistic approach. Due to the possibility of male influence to recall soccer information (FIFA World Cup text), this factor was controlled in the analysis.

Intentionality was shown to be a factor that improves memory performance in the studied sample only on day 2. The within-subjects analysis indicates that the IL group recalled more information on day 2 than of day 7. This occurred without the interaction with age, gender, and education in these results.

Additionally, there were no differences when comparing memory performance at baseline and on days 2 and 7 in the TEG group, showing that repeating a test may stabilize the information acquired over the days. This result is related to studies such as a systematic review which indicates that the testing effect proves to be more effective than re-studying the material or answering a multiple choices test^33^ and to a study indicating that tests with feedback have greater retention of information over time.^24^

The between-subjects results showed an effect of schooling and gender in the IL group. When the performances of men and women were compared on each day, there were no differences on day 7, only on day 2, with men recalling more information. In the TEG, men also showed better results, but this occurred at the baseline and on days 2 and 7. The results are supported by the literature, especially when referring to a content closer to the relevant target, in this case, for men (FIFA World Cup text). More specifically, the difference between men and women was found in recall, as they differ in the types of cognitive strategies for processing information and in the skills of recording.^32^ The effect of schooling has been observed previously in intentional learning situations, in which participants with high levels of education presented better cognitive performance.^34^ Schooling has been associated with better cognitive performance, as well as a proxy for cognitive reserve.^35^

The effect of intentionality occurred 2 days after the exposure session, however, it does not remain when evaluated 7 days later, suggesting that the effect of intention to remember is time-dependent. It has long been known that the intention to learn has a positive effect on subsequent recall, and most studies assessing the intention to learn evaluated retrieval on the same day as the encoding, *i.e*., from minutes to hours,^15^ ^,^ ^16^ ^,^ ^17^ ^,^ ^20^ ^,^ ^21^ or at most for two days.^18^ ^,^ ^19^ Our study is the first one to investigate the lastingness of the effect on intentionality up to seven days. These results indicate intentionality can be used as a strategy to improve memory for short-term objectives. Because the ability to retain more intentional information also depends on a more extensive neural network.^36^ ^,^ ^37^

Intentional learning is an induced motivation, whose basis relies on the dopaminergic system.^38^ Impairment of intentional learning was observed in patients with Parkinson disease, which was attributed to dopamine depletion.^38^ One possible explanation for the influence of motivation on memory retrieval could be related to the dopaminergic system.^39^ This system is involved not only with motivation but also with attentional processes,^40^ which are necessary for memory functioning.^37^ The enhancement of the dopaminergic system, through motivation, could improve memory retrieval. In fact, a positive effect of methylphenidate (a drug widely believed to act on the enhancement of dopaminergic synapses) has been already demonstrated on incidental and formal memories.^31^

Unexpectedly, there was no age effect on memory performance. This finding may be explained by two possibilities. First, the memory task used in this study does not offer a high level of difficulty to differentiate memory performance between different age groups. Episodic memory tests may vary according to the degree of organization inherent in the material to be memorized and the mode of reproduction. Tasks requiring the recall of semantically related materials or stories (*e.g.* logical memory tasks) are thought to be easier that those requiring the recall of semantically unrelated materials (*e.g*. list of words such as the Rey Auditory Verbal Learning Test - RAVLT).^41^ Secondly, older adults tend to present deficits in associative memory tests which are more pronounced under intentional conditions.^10^ The age-related associative deficit indicates older adults have increased difficulty in using self-initiated processes (to bind pieces of information together). In our study, we used a memory test in which participants were required to recall pieces of information and not to associate information. There are some limitations that should be pointed out. First, the study was not designed in such a way that analyses between groups could be carried out. Also, the study used a text about the FIFA World Cup, which benefited the impact of gender, since it is usually a male preference subject.

In conclusion, the intention to remember improved the recall of information over a short period of days, but this effect did not last for a week. Future studies should investigate the benefit and the persistence of intentionality in the memory of patients with cognitive impairment, as well as the use of texts with neutral content. In addition, new studies that insert clinical data (such as analysis of the dopaminergic system) together with motivation may be a factor that influences recovery.
